# Lean body mass mediates the sex dimorphism in cardiac and large arterial elastances

**DOI:** 10.1016/j.mmr.2026.100016

**Published:** 2026-03-25

**Authors:** Xin-Yu Liu, Mei-Han Guo, David Montero

**Affiliations:** School of Public Health, Faculty of Medicine, Hong Kong University, Hong Kong 999077, China; Department of Medicine, Beth Israel Deaconess Medical Center, Harvard Medical School, Boston, MA 02115, USA; School of Public Health, Faculty of Medicine, Hong Kong University, Hong Kong 999077, China; Department of Medicine, School of Clinical Medicine, Hong Kong University, Hong Kong 999077, China; Libin Cardiovascular Institute of Alberta, University of Calgary, Alberta T2N 4N1, Canada

**Keywords:** Lean body mass (LBM), Cardiac elastances, Arterial elastance, Systemic vascular resistance (SVR), Female-prone cardiac concentric remodeling, Sex dimorphism

Dear Editor,

The female heart is subjected to higher pressure than the male heart. Up to approximately 50% increments in cardiac and large arterial elastances (defined as pressure per unit of volume) have been observed in healthy women relative to men [1], [2], [3]. Consequently, cardiac and large arterial elastances represent a prominent sex dimorphism in the circulatory system. This plausibly contributes to the higher prevalence of left ventricular (LV) concentric hypertrophy in women than in men [4]. Moreover, such a sex dimorphism may underpin the increased risk of heart failure with preserved ejection fraction (HFpEF) in women, a disease entailing a 5-year mortality rate >50% [5].

The question arises: what mechanism explains the sex dimorphism in cardiac and large arterial elastances? Recent studies have disclosed strong relationships between body composition, specifically lean body mass (LBM), and the structure and function of the circulatory system [6], [7], [8]. Among potential causal links, the amount of LBM, which is substantially lower (–30%) in women than in men [6], [7], [8], determines the resistance that the LV must overcome to eject blood into the systemic circulation. The lower the LBM, the higher the systemic vascular resistance (SVR) [6], [7], [8], also known as cardiac afterload. Hence, the female LV, which is chronically exposed to elevated afterload, becomes relatively hypertrophied and stiffer. This, combined with the smaller LV volume in women compared with men, even when normalized by body size [9], may hypothetically result in augmented cardiac and large arterial elastances in the former. The present study aimed to test the hypothesis that LBM, primarily, and SVR, secondarily, explain the sex dimorphism in cardiac and large arterial elastances. LBM and SVR are amenable to modification, thus potentially being effective therapeutic targets in the female population [5].

Detailed methods are presented in **Additional file 1.** General characteristics, cardiac and large arterial elastances, body weight, and composition in women and men are presented in Additional file 1: Table S1**.** A total of 303 women (*n=*149) and men (*n=*154) matched by age [(43.7±18.6) vs. (45.1±18.5) years, *P=*0.525], physical activity [total moderate-to-vigorous physical activity (MVPA)=(5.3±3.5) vs. (5.8±3.5) h/week, *P=*0.208], and endurance-specific MVPA [(MVPA-END)=(4.6±3.4) vs. (5.0±3.5) h/week, *P=*0.420] were recruited via paper and digital advertisements. Women had lower BMI [(21.7±2.5) vs. (23.4±3.0) kg/m^2^] and BSA [(1.58±0.13) vs. (1.84±0.15) m^2^] compared with men (*P<*0.001 for both). Peak oxygen consumption (VO_2peak_) and arterial blood pressures [systolic blood pressure (SBP) and diastolic blood pressure (DBP)] were reduced in women compared with men (*P*≤0.006). Cardiac elastances LV [end-systolic elastance (Ees), diastolic elastance (Ed)], and large arterial elastance (Ea) were augmented in women relative to men (*P<*0.001).

In regression analyses, sex (men: 1; women: 2) was positively associated with Ees (*β*=0.52, *P<*0.001), Ed (*β*=0.84, *P<*0.001), and Ea (*β*=0.78, *P<*0.001). Regarding body composition, women presented with lower total LBM (*P<*0.001) and higher total body fat (*P<*0.001) than men (Additional file 1: Table S2**).**

The mediation effects of body weight, body fat, total LBM, and leg LBM variables in the relationship of sex with cardiac and large arterial elastances are illustrated in Fig. 1. Body weight was not a mediator in the relationship of sex with Ees. Body weight partially mediated the relationship of sex with Ed (*β*=0.22) and Ea (*β*=0.29); a direct effect of sex on Ed and Ea was significant (*P*<0.001) (Fig. 1**;** Additional file 1: Table S2). Body fat was not a mediator in the relationship between sex and Ed. Body fat had a minor partial mediation effect in the relationship of sex with Ees (*β*=0.07) and Ea (*β*=0.05); a direct effect of sex on Ees and Ea was significant (*P<*0.001) (Fig. 1**;** Additional file 1: Table S2). Total LBM was a mediator in the relationship of sex with Ees, Ed, and Ea. Total LBM partially mediated the relationship of sex with Ed (*β*=0.49); a direct effect of sex on Ed was present (*P=*0.029). Total LBM mediated the relationship of sex with Ees (*β*=0.29), but this effect did not differ from the non-significant direct effect of sex on Ees (*P=*0.173). Total LBM completely mediated the relationship of sex with Ea (*β*=0.74); There was no direct effect of sex on Ea (*P=*0.812) (Fig. 1**;** Additional file 1: Table S2). Similar mediation effects were observed for total and leg LBM (Fig. 1**;** Additional file 1: Table S2). Given the complete mediation of total or leg LBM in the relationship of sex with Ea, the potential secondary mediation effect of SVR in the mediation of total or leg LBM in the relationship of sex and Ea was analyzed and presented in Additional file 1: Figs. S1 and S2. SVR mediated the mediation effect of total LBM or leg LBM in the relationship of sex with Ea (*β*≥0.46) (Additional file 1: Fig. S2); a primary mediation effect of total LBM or leg LBM on Ea was still present but with lesser magnitude (*β*≥0.28) (Additional file 1: Fig. S2**;** Table S3).

**Fig. 1:**
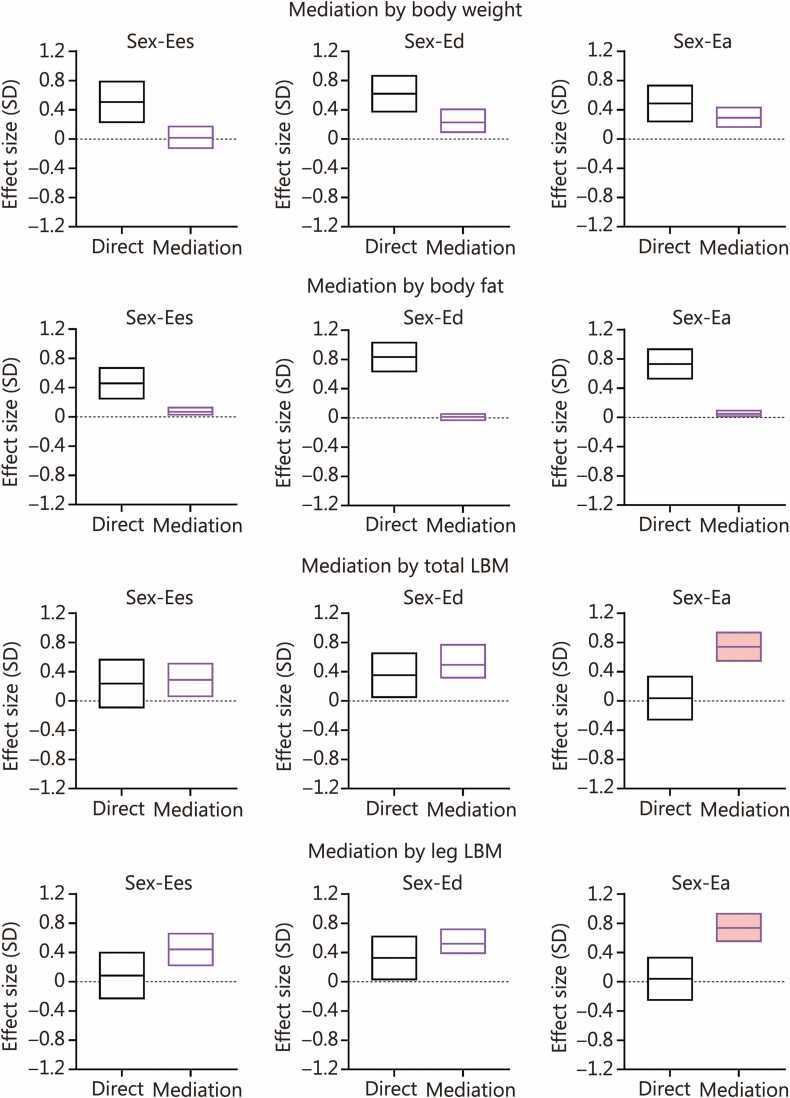
Mediation effect of body composition in the relationship of sex with cardiac and large arterial elastances. The 95% confidence intervals (CI) of the effect sizes of direct and mediation effects are illustrated by floating bars. The standardized regression coefficients (*β*) are depicted by the middle horizontal line in the floating bars. *β* represents the change in SD units in the dependent variable (cardiac or large arterial elastances: Ees, Ed, or Ea) per unit change in sex. Sex is defined as a binary categorical variable (men: 1; women: 2). When the floating bar does not cross and/or comprise the zero value (represented by the discontinued horizontal line), the effect size is significant (*P*<0.05). Partial mediation effects are graphically illustrated by open (blank) floating bars, whereas complete mediation effects are illustrated by filled (red) floating bars. Ea. Arterial elastance; Ed. Diastolic elastance; Ees. End-systolic elastance; LBM. Lean body mass; SD. Standard deviation.

This study reveals the potential role of body composition in the prominent sex dimorphism in cardiac and large arterial elastances. Women presented with greater cardiac (Ees, Ed) and large arterial (Ea) elastances compared with age- and physical activity-matched men (Additional file 1: Table S1). Neither body weight nor body fat mediated at all or had a mediation effect greater than the direct effect in the relationship of sex with Ees, Ed, and Ea. In contrast, total and leg LBM 1) partially mediated the relationship of sex with Ees and Ed, and 2) completely mediated the relationship of sex with Ea (Fig. 1**;** Additional file 1: Table S1). Accordingly, total and leg LBM fully explained the sex difference in large arterial elastance. The addition of SVR, as a secondary mediator, enhanced the mediation effect of total and leg LBM. Therefore, the increased large arterial elastance in women relative to men can be explained by augmented SVR associated with lower LBM in the former. This concurs with the hypothesis that LBM determines the degree of peripheral vascular conductance, which reflects upstream, as cardiac afterload, eliciting chronic structural and biomechanical adaptations in large elastic arteries and the LV [6], [7], [8]. This upstream-oriented mechanistic rationale is reinforced by the fact that the mediation effect of LBM was more pronounced for sex differences in large arterial than cardiac (systolic, diastolic) elastances, the latter being primarily influenced by cardiac preload [10].

Taken together, markedly elevated cardiac and large arterial elastances in women are expected owing to their substantially reduced LBM and thereby increased SVR relative to men. Yet, BMI or BSA were not included as covariates in the mediation analyses; therefore, residual confounding in adiposity and body size can not be fully excluded. Moreover, given the observational nature of this study, we are unable to determine the underlying biological basis. Potential molecular mechanisms contributing to the present sex dimorphism will be substantiated in future studies. Currently established lifestyle and/or pharmacological interventions targeting LBM may have the potential to reduce cardiac and notably large arterial elastances in the female population.

## Abbreviations

DBP: Diastolic blood pressure
Ea: Arterial elastance
Ed: Diastolic elastance
Ees: End-systolic elastance
HfpEF: Heart failure with preserved ejection fraction
LBM: Lean body mass
LV: Left ventricular
MVPA: Moderate-to-vigorous physical activity
MVPA-END: Endurance-specific MVPA
SBP: Systolic blood pressure
SVR: Systemic vascular resistance
VO_2peak_: Peak oxygen consumption

## Ethics approval and consent to participate

Not applicable.

## Funding

This work was supported by the Research Grant Council of Hong Kong-ECS (106210224) and SF (104006024).

## Data Availability

All data associated with this study will be available upon reasonable request to the corresponding author.
