# Supplementary material for: Lean body mass mediates the sex dimorphism in cardiac and large arterial elastances
Source: Mil Med Res. 2026 Mar 25;13(1):100016. doi: 10.1016/j.mmr.2026.100016 (PMC13127128; doi:10.1016/j.mmr.2026.100016)
Supplement: Supplementary file 1 — Supplementary material [file mmc1.pdf]

## Methods

### Study participants and ethical approval

This observational study was performed in the University of Hong Kong (Hong Kong, China) and the University of Calgary (Alberta, Canada) from January 1st, 2019, to December 20th, 2024. A total of 303 women and men were recruited throughout the adult lifespan (18–78 years old), matched by age and physical activity. Inclusion criteria comprised healthy status according to health-clinical questionnaires and resting electrocardiogram (ECG)-echocardiography screening, absence of current medical symptoms/medication, and no history of chronic disease. Exclusion criteria comprised regular smoking (≥1 cigarette/d) and obesity [body mass index (BMI) ≥30]. The study was approved by the Institutional Review Board of the University of Hong Kong (UW 21-401/22-025)-Conjoint Health Research Ethics Board (REB18-1654) of the University of Calgary and conducted in accordance with the Declaration of Helsinki. Before the start of the measurements, informed oral and written consents were obtained from the participants.

### Measurements

In accordance with standard exercise testing procedures, the participants were instructed to avoid strenuous exercise, alcohol, and caffeine for 24 h before they visited the laboratory. The participants were asked to void their bladder in the restroom before starting the measurements. Fluid intake was recorded for the last 5 h prior to their visit to the laboratory.

#### *Body composition*

Lean body mass (LBM), fat, and bone mass in total and per body region (legs, arms, trunk) were determined via dual-energy X-ray absorptiometry (DXA) (Hologic QDR 4500) following best practice recommendations [1]. The participants were instructed to sit down in the middle of the scanning table. Subsequently, the participants lay supine with their spine aligned with the impressed longitudinal midline of the scanning table. Their arms were placed along their side with the palms pronated to standardize the scanned area. A fixed space was kept between wrists and trunk (5 cm) and between ankles (10 cm) to facilitate segmentation in regional assessments.

#### *Cardiovascular structure and function*

Cardiac structure and function at rest were assessed via high-resolution ultrasound (Mindray Medical M9). Apical four-chamber and two-chamber cine-loops were recorded and assessed offline via high-resolution ultrasound. Following the American Society of Echocardiography and the European Association of Cardiovascular Imaging recommendations, cardiac chamber quantification, including left ventricular (LV) end-diastolic and end-systolic volumes (LVEDV, LVESV), was performed using

the modified Simpson method (biplane method of disks) by tracing the endocardial border of the LV in apical four-chamber and two-chamber views at end-diastole and end-systole [2,3]. The difference between LVEDV and LVESV provided stroke volume (SV). Cardiac output (Q) was calculated as the product of SV and heart rate. Resting arterial blood pressures (systolic blood pressure (SBP), diastolic blood pressure (DBP), mean arterial pressure (MAP) were determined via the volume-clamp method in the middle finger of the left hand, calibrated to brachial artery pressure and positioned at the heart height level (CNAP® Monitor 500 HD, CNSystems). Systemic vascular resistance (SVR) was defined as the ratio of MAP and Q [4,5]. To assess LV function, transmitral inflow velocities were determined by pulsed-wave Doppler, with the sample volume placed between the mitral leaflet tips in the apical four-chamber view. The peak inflow velocities during early (E) and late (A) diastole were measured. In addition, myocardial tissue e' and a' velocities were measured via tissue Doppler imaging (TDI) in the septal ventricular wall adjacent to the mitral annulus. The ratio of E and septal e' (E/e'), which reflects LV filling pressure, was determined according to established guidelines [2,3]. With respect to cardiac and arterial elastances, LV end-systolic elastance [Ees=(0.9×SBP)/LVESV], LV diastolic elastance [Ed=(E/e')/SV], and large arterial elastance [Ea=(0.9×SBP)/SV] were determined conforming to consensus recommendations [6,7].

Cardiorespiratory fitness, represented by pulmonary peak oxygen $O_2$ consumption ($VO_{2peak}$), was assessed via incremental cycle ergometry, as previously described [5]. $VO_2$, $CO_2$ output, and ventilation were recorded via a mixing chamber system (KORR Medical, USA) throughout an incremental exercise test. Following a warm-up period at 10–30 W, the workload was progressively increased by 10–30 W increments every 50 s until exhaustion was reached in the recommended total duration of 6–9 min [8]. Calibration of the gas analyzers and the flowmeter was conducted before each test. Values were averaged over 15 s following current recommendations [9].

**Statistical analysis**

Statistical analyses were performed with SPSS 26.0. Variables were reported as mean±standard deviation (SD). The relationship between sex and cardiac and large arterial elastances was assessed using linear regression analyses. Body composition variables were included as potential mediators in mediation analyses conducted using the PROCESS macro for SPSS (version 4.2 beta; https://www.processmacro.org/index.html). Provided that a body composition variable fully mediated the relationship of sex with cardiac or large arterial elastances (primary mediation), the potential secondary mediation of SVR in the primary mediation was additionally determined. The direct effect represents the portion of the association that remains independent of the mediation by a body composition variable or SVR. A mediation effect can be 1) partial, in which the relationship between sex and the elastance variable is partially explained by direct and mediation (indirect) effects, or 2)

complete, i.e., the relationship between sex and the elastance variable is fully explained by the mediation effect (which frequently differs from the direct effect). Sex is defined as a binary categorical variable (men: 1; women: 2). Accordingly, the reference group is men, and a positive standardized regression coefficient ($\beta$) represents an increased effect on the elastance variable in women relative to men. The lower (LLCI)/upper (ULCI) levels of 95% confidence intervals (CI) from 5000 bootstrap samples were obtained for each potential mediation effect.

**Table S1** Characteristics of study participants (mean±SD)

| **Variables** | **Women (*n* = 149)** | **Men (*n* = 154)** | ***P*-value** |
|---|---|---|---|
| General characteristics | | | |
| Age (year) | 43.7±18.6 | 45.1±18.5 | 0.525 |
| Height (cm) | 161.0±6.5 | 173.7±7.0 | <0.001 |
| Weight (kg) | 56.3±7.9 | 70.7±10.5 | <0.001 |
| BMI ($kg/m^2$) | 21.7±2.5 | 23.4±3.0 | <0.001 |
| BSA ($m^2$) | 1.58±0.13 | 1.84±0.15 | <0.001 |
| SBP (mmHg) | 114.0±20.4 | 125.0±15.5 | <0.001 |
| DBP (mmHg) | 72.1±12.5 | 75.9±11.3 | 0.006 |
| MVPA (h/week) | 5.3±3.5 | 5.8±3.5 | 0.208 |
| MVPA-END (h/week) | 4.6±3.4 | 5.0±3.5 | 0.420 |
| $VO_{2peak}$ [ml/(min·kg)] | 31.6±8.2 | 40.9±10.9 | <0.001 |
| Cardiac & large arterial elastances | | | |
| Ees (mmHg/ml) | 6.07±2.86 | 4.74±1.99 | <0.001 |
| Ed ($ml^{-1}$) | 0.07±0.03 | 0.05±0.01 | <0.001 |
| Ea (mmHg/ml) | 1.26±0.32 | 1.02±0.23 | <0.001 |
| SVR | 1408.1±366.0 | 1146.5±261.6 | <0.001 |
| Body composition | | | |
| Total BMC (kg) | 1.90±0.32 | 2.53±0.39 | <0.001 |
| Total LBM (kg) | 38.5±5.3 | 54.5±7.7 | <0.001 |
| Total body fat (kg) | 16.7±4.4 | 14.6±4.8 | <0.001 |
| Total body fat (%)* | 29.0±5.4 | 20.2±4.8 | <0.001 |
| Leg LBM (kg) | 12.8±2.0 | 18.6±2.8 | <0.001 |
| Leg fat (kg) | 6.7±1.7 | 5.0±1.6 | <0.001 |
| Leg fat (%)* | 32.9±5.9 | 20.3±5.0 | <0.001 |
| Arm LBM (kg) | 3.3±0.8 | 6.0±1.5 | <0.001 |
| Arm fat (kg) | 1.9±0.6 | 1.7±0.6 | <0.001 |
| Arm fat (%)* | 35.1±7.1 | 20.8±5.6 | <0.001 |
| Trunk LBM (kg) | 18.9±2.8 | 25.8±4.1 | <0.001 |
| Trunk fat (kg) | 6.9±2.6 | 6.6±2.8 | 0.345 |
| Trunk fat (%)* | 25.7±6.5 | 19.5±5.8 | <0.001 |

*Fat percentage was calculated as kg of fat divided by kg in total (or per body region) and multiplied by 100. BMC. Bone mineral content; BMI. Body mass index; BSA. Body surface area; DBP. Diastolic blood pressure; Ea. Large arterial elastance; Ed. Left ventricular diastolic elastance; Ees. Left ventricular end-systolic elastance; LBM. Lean body mass; MVPA. Moderate-to-vigorous physical activity; MVPA-END. Moderate-to-vigorous physical activity comprising endurance exercise; SBP. Systolic blood pressure; $VO_{2peak}$. Peak oxygen consumption; SVR. Systemic vascular resistance

**Table S2** Mediation effect of body composition in the relationship of sex with cardiac and large arterial elastances

| **Mediators** | | ***β*** | **95% CI** | ***P*-value** |
|---|---|---|---|---|
| Body weight | | | | |
| Sex-Ees | Direct | 0.51 | 0.22−0.80 | <0.001 |
| | Mediation | 0.02 | −0.14 to 0.18 | > 0.050 |
| Sex-Ed | Direct | 0.62 | 0.36−0.88 | <0.001 |
| | Mediation | 0.22 | 0.08−0.42 | <0.050 |
| Sex-Ea | Direct | 0.49 | 0.23−0.74 | <0.001 |
| | Mediation | 0.29 | 0.15−0.44 | <0.050 |
| Body fat | | | | |
| Sex-Ees | Direct | 0.46 | 0.23−0.68 | <0.001 |
| | Mediation | 0.07 | 0.02−0.14 | <0.050 |
| Sex-Ed | Direct | 0.83 | 0.62−1.04 | <0.001 |
| | Mediation | 0.01 | −0.05 to 0.07 | > 0.050 |
| Sex-Ea | Direct | 0.73 | 0.52−0.94 | <0.001 |
| | Mediation | 0.05 | 0.00−0.10 | <0.050 |
| Total LBM | | | | |
| Sex-Ees | Direct | 0.24 | −0.10 to 0.58 | 0.173 |
| | Mediation | 0.29 | 0.05−0.52 | <0.050 |
| Sex-Ed | Direct | 0.35 | 0.04−0.66 | 0.029 |
| | Mediation | 0.49 | 0.31−0.78 | <0.050 |
| Sex-Ea | Direct | 0.04 | −0.27 to 0.34 | 0.812 |
| | Mediation | 0.74 | 0.53−0.95 | <0.050 |
| Leg LBM | | | | |
| Sex-Ees | Direct | 0.08 | −0.24 to 0.41 | 0.620 |
| | Mediation | 0.44 | 0.21−0.67 | <0.050 |
| Sex-Ed | Direct | 0.32 | 0.02−0.63 | 0.038 |
| | Mediation | 0.52 | 0.38−0.72 | <0.050 |
| Sex-Ea | Direct | 0.04 | −0.26 to 0.34 | 0.795 |
| | Mediation | 0.74 | 0.54−0.94 | <0.050 |

Direct path: direct effect of sex on cardiac and Ea after accounting for body composition mediators. Mediation path: indirect effect of sex on cardiac and Ea mediated by body composition. *β*. Standardized regression coefficient; CI. Confidence interval; Ea. Arterial elastance; Ed. Diastolic elastance; Ees. End-systolic elastance

**Table S3** Mediation effect of total or leg lean body mass (LBM) excluding (mediation-1) or including (mediation-2) the secondary mediation effect of systemic vascular resistance (SVR) in the relationship of sex with large arterial elastance

| **Mediators** | | ***β*** | **95% CI** | ***P*-value** |
|---|---|---|---|---|
| Total LBM and SVR | | | | |
| Sex-Ea | Direct | −0.05 | −0.26 to 0.16 | 0.627 |
| | Mediation 1 | 0.28 | 0.13−0.45 | <0.050 |
| | Mediation 2 | 0.46 | 0.30−0.62 | <0.050 |
| Leg LBM and SVR | | | | |
| Sex-Ea | Direct | −0.05 | −0.26 to 0.15 | 0.622 |
| | Mediation 1 | 0.28 | 0.11−0.46 | <0.050 |
| | Mediation 2 | 0.45 | 0.30−0.62 | <0.050 |

Direct path: direct effect of sex on Ea after accounting for the mediators (total or leg LBM and SVR). Mediation 1 path: indirect effect of sex on Ea mediated by total or leg LBM excluding SVR. Mediation 2 path: indirect effect of sex on Ea mediated by total or leg LBM, including SVR. *β*. Standardized regression coefficients; CI. Confidence intervals; Ea. Arterial elastance

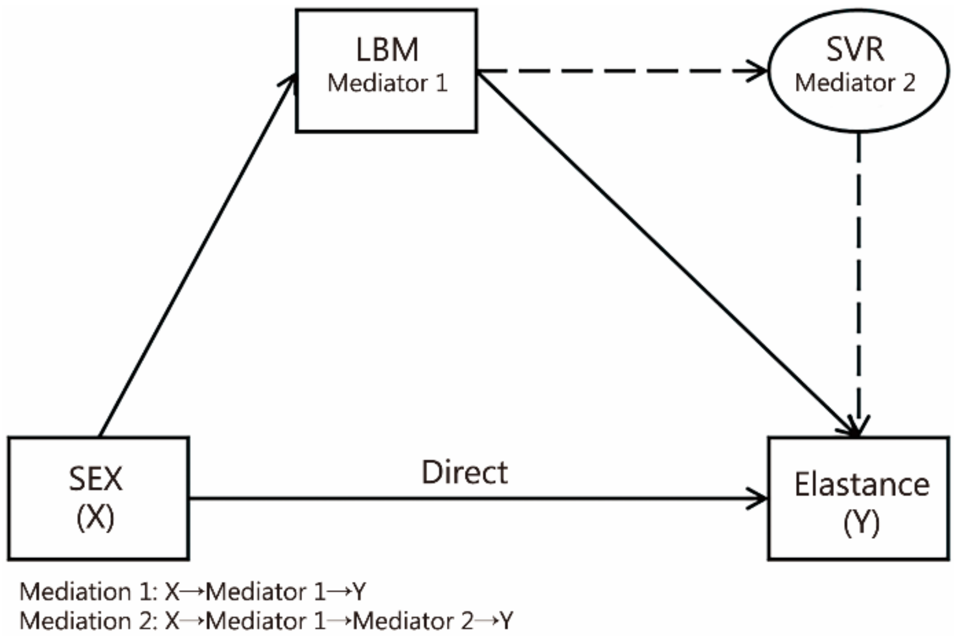


**Fig. S1** Diagram of the main evaluated mediation pathways between sex and cardiac/large arterial elastances. LBM. Lean body mass; SVR. Systemic vascular resistance; Y. Outcome variable; X. Independent variable (sex)

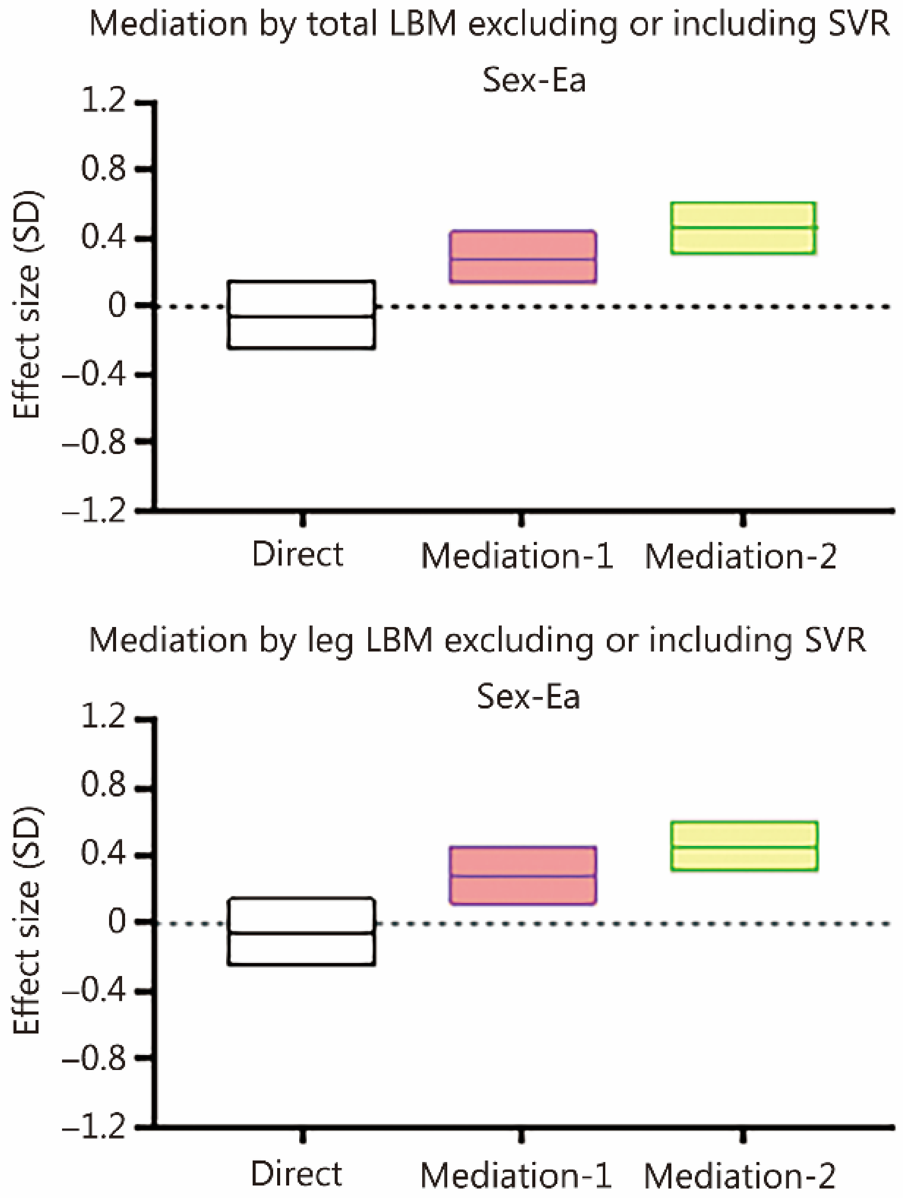


**Fig. S2** Mediation effect of total or leg lean body mass (LBM) excluding (mediation-1) or including (mediation-2) the secondary mediation effect of systemic vascular resistance (SVR) in the relationship of sex with large arterial elastance. The 95 % confidence intervals (CI) of the effect sizes of direct and mediation effects are illustrated by floating bars, with the standardized regression coefficient (*β*) depicted by the middle horizontal line. *β* represents the change in standard deviation (SD) units in the dependent variable (Ea, large arterial elastance) per unit change in sex. When the floating bar does not cross and/or comprise the zero value (represented by the discontinued horizontal line), the effect size is significant ($P<0.05$). A mediation effect can be 1) partial, in which the relationship is partially explained by direct and mediation (indirect) effects, or 2) complete, i.e., the relationship between sex and large arterial elastance is fully explained by the mediation effect, which significantly differs from the direct effect. Partial mediation effects are graphically illustrated by open (blank) floating bars, whereas complete mediation effects are illustrated by filled (red or yellow) floating bars

## References


1. Lewiecki EM, Binkley N, Morgan SL, Shuhart CR, Camargos BM, Carey JJ, *et al*. Best practices for dual-energy X-ray absorptiometry measurement and reporting: international society for clinical densitometry guidance. J Clin Densitom. 2016;19(2):127-40.
2. Lang RM, Badano LP, Mor-Avi V, Afilalo J, Armstrong A, Ernande L, *et al*. Recommendations for cardiac chamber quantification by echocardiography in adults: an update from the American Society of Echocardiography and the European Association of Cardiovascular Imaging. Eur Heart J Cardiovasc Imaging. 2015;16(3):233-70.
3. Pellikka PA, Nagueh SF, Elhendy AA, Kuehl CA, Sawada SG, American Society of E. American Society of Echocardiography recommendations for performance, interpretation, and application of stress echocardiography. J Am Soc Echocardiogr. 2007;20(9):1021-41.
4. Diaz-Canestro C, Pentz B, Sehgal A, Montero D. Sex dimorphism in cardiac and aerobic capacities: the influence of body composition. Obesity (Silver Spring). 2021;29(11):1749-59.
5. Diaz-Canestro C, Pentz B, Sehgal A, Yang R, Xu A, Montero D. Lean body mass and the cardiovascular system constitute a female-specific relationship. Sci Transl Med. 2022;14(667):eabo2641.
6. Ikonomidis I, Aboyans V, Blacher J, Brodmann M, Brutsaert DL, Chirinos JA, *et al*. The role of ventricular-arterial coupling in cardiac disease and heart failure: assessment, clinical implications and therapeutic interventions. A consensus document of the European Society of Cardiology Working Group on Aorta & Peripheral Vascular Diseases, European Association of Cardiovascular Imaging, and Heart Failure Association. Eur J Heart Fail. 2019;21(4):402-24.
7. Redfield MM, Jacobsen SJ, Borlaug BA, Rodeheffer RJ, Kass DA. Age- and gender-related ventricular-vascular stiffening: a community-based study. Circulation. 2005;112(15):2254-62.
8. Astorino TA, Rietschel JC, Tam PA, Taylor K, Johnson SM, Freedman TP, *et al*. Reinvestigation of optimal duration of $VO_{2max}$ testing. J Exerc Physiol. 2004;7(6):1-8.
9. Martin-Rincon M, Calbet JAL. Progress update and challenges on $VO_{2max}$ testing and interpretation. Front Physiol. 2020;11:1070.
